# Short description of an alternative simplified method for screening recombinant clones within the "AdEasy-System" by Duplex-PCR

**DOI:** 10.1186/1472-6750-5-1

**Published:** 2005-01-11

**Authors:** Dalibor Antolovic, Moritz Koch, Inga Bohlmann, Peter Kienle, Markus Büchler, Jürgen Weitz

**Affiliations:** 1 Department of Surgery, University of Heidelberg; Heidelberg, Germany; 2 Department of Gynecology and Obstetrics, University of Hamburg, Hamburg, Germany

## Abstract

**Background:**

Recombinant adenoviral vectors are highly efficient for in vitro and in vivo gene delivery. They can easily be produced in large numbers, transduce a wide variety of cell types and generate high levels of transgene expression.

The AdEasy system is a widely used system for generating recombinant adenoviral vectors, which are created with a minimum of enzymatic manipulations and by employing homologous recombination in E. coli.

In this paper we describe an alternative simplified method for screening recombinant DNA within the AdEasy system. This Duplex-PCR-method is independent of the transgene or insert and can be used for the complete AdEasy system. It is characterized by a simple standard protocol and the results can be obtained within a few hours. The PCR is run with two different primer sets. The primers KanaFor and KanaRev hybridizise with the Kanamycin resistence gene and AdFor and AdRev detect the adenoviral backbone. In case of recombinant clones, two diagnostic fragments with a size of 384 bp and 768 bp are generated.

**Results:**

The practicability of this method was verified with three different transgenes: Cytosin Deaminase (AdCD), p53 (Adp53) and Granulocyte Macrophage Colony Stimulating Factor (AdGM-CSF). Recombinant clones are indicated by two diagnostic fragments and are then suitable for further processing.

**Conclusion:**

In summary, the presented protocol allows fast detection of recombinants with an easy technique by minimizing the amount of necessary steps for generating a recombinant adenovirus. This method is time sparing and cost-effective.

## Background

Recombinant adenoviral vectors are highly efficient for in vitro and in vivo gene delivery. They can easily be produced in large numbers, transduce a wide variety of cell types and generate high levels of transgene expression. The AdEasy system is a widely used, simplified system for generating recombinant adenoviral vectors, which are created with a minimum of enzymatic manipulations and by employing homologous recombination in E. coli [1]. The system consists of two adenoviral backbone vectors (pAdEasy-1 with deleted E1/E3 and pAdEasy-2 with deleted E1/E3/E4) and four different shuttle vectors (pShuttle, pShuttle-CMV, pAdTrack, pAdTrack-CMV), into which the desired transgenes are inserted. The polylinkers are surrounded by adenoviral sequences that allow homologous recombination with adenoviral backbone plasmids in E. coli. The shuttle vectors differ by partly carrying a cytomegalievirus (CMV) promoter and GFP as a tracer all of which contain a kanamycin resistance gene. Therefore, the various components can easily be combined depending on the desired purpose. In this paper we describe a simplified and easy method for screening recombinant DNA within the AdEasy system. This Duplex-PCR-method is independent of the transgene or insert and can be used for the complete AdEasy-System. It is characterized by a simple standard protocol and the results can be obtained within a few hours. The PCR is run with two different primer sets. The primers KanaFor and KanaRev hybridizise with the Kanamycin resistence gene and AdFor and AdRev detect the adenoviral backbone. In case of recombinant clones, two diagnostic fragments with a size of 384 bp and 768 bp are generated.

## Methods

The presented Duplex-PCR is performed as follows: After Co-transformation of the Pme I-digested shuttle vector with the adenoviral backbone plasmid to E. coli (BJ 5183) and plating on agar (selection on kanamycin), half of the overnight grown colonies are picked and used directly as template for the colony-PCR. The other half of the colony is used for inoculation with LB-kanamycin and then incubation at 37°C. Only the positive, recombinant clones which have been detected by PCR are grown overnight, a minipreparation of DNA is then performed the next morning.

The PCR is run using two different primer sets. The primers KanaFor (5' CAA GAT GGA TTG CAC GCA GG 3') and KanaRev (5'AAG GCG ATA GAA GGC GAT GC 3') hybridize to the Kanamycin resistence gene and AdFor (5'GGC TGC TCT GCT CGG AAG AC 3') and AdRev (5'GGC ATA CGC GCT ACC CGT AG 3') detect the adenoviral backbone.

**Figure 1:**
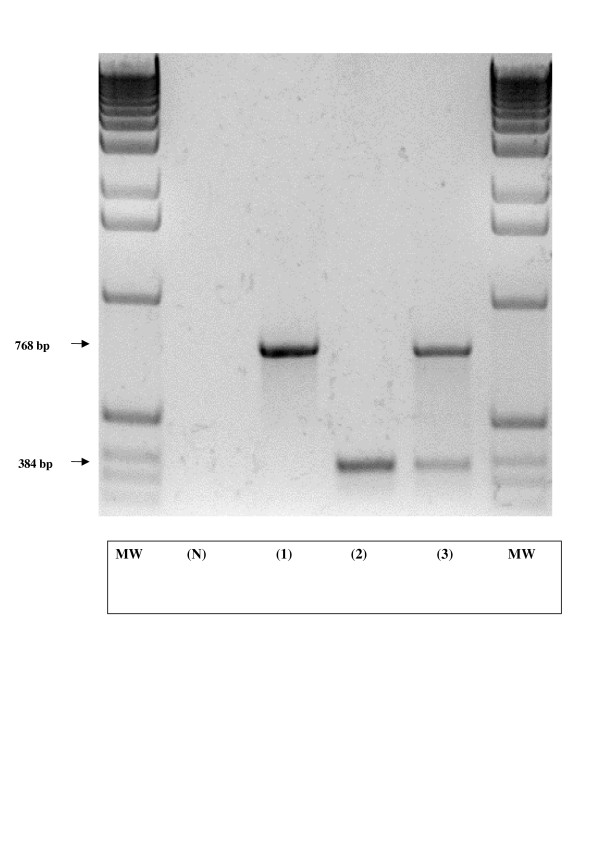
Duplex-PCR Gel electrophoresis for PCR products from Colony Duplex PCR with two primer sets AdFor/AdRev and KanaFor/KanaRev on AdEasy system generating two diagnostic fragments of 384 bp and 768 bp (see arrows) after homologous recombination.(N) negative control; (1) pShuttle/CD; (2) pAdEAsy 1; (3) pAdEasy/CD, e.g. recombinant clone

The optimised concentrations of the components for the Duplex-PCR were as follows: 2, 5 mM MgCl_2_, 100 mM dNTPs and 0,2 Units Taq polymerase. The bacteria are denatured at 95°C for 10 minutes. The PCR-products are amplified by 40 cycles of annealing at 58°C (30 sec), extension at 72°C (30 sec) and denaturation at 94°C (30 sec). In our experience, this procedure produced the best results without generating false positive clones (Figure 1).

PCR products are analyzed by agarose gel electrophoresis, half of the reaction volume (25 μl) is size fractionated with 80 V for 1 h in 1% agarose in the presence of ethidium bromide and the resulting bands visualized with ultraviolet illumination.

The DNA obtained by small scale alkali lysis from the recombinants is then extracted twice with a phenol-chloroform protocol, precipitated and carefully resuspended in 20 μl RNAse-free water. The construct is linearized with PacI and directly transfected into 911 cells, which are monitored for cytopathic effects, i.e. production of recombinant adenoviruses. The cytopathic effect is usually seen within 5 to 10 days. The expression of the transgene is confirmed by Western blot analysis. The practicability of our procedure was verified with three different transgenes: Cytosin Deaminase (AdCD), p53 (Adp53) and Granulocyte Macrophage Colony Stimulating Factor (AdGM-CSF).

## Results and discussion

The conventional way of screening for recombinants after Co-transformation of the linearized shuttle vector with the adenoviral backbone vector in E. coli is by plating on LB/kanamycin, growing the bacteria overnight, then picking the colonies and growing them again for 10–15 hours. Minipreps are then performed and the size is evaluated on agarose gels. A restriction digest with three different restriction enzymes is then done and finally again another agarose gel is run. This is a relatively time-consuming and laborious procedure, which takes about 2 days.

In contrast, the presented alternative protocol allows fast detection of recombinants with a simplified technique by minimizing the amount of necessary steps for generating a recombinant adenovirus. The method is time sparing and cost-effective. In our experience, the above described protocol showed no problems with false negative clones. After optimisation of the PCR protocol, we were able to run the conventional screening method (e.g. by restriction digest) for recombinant clones at the same time as the presented simplified PCR. We found no differences in regard to the final results for the two methods, but it has to be kept in mind that only a limited number of recombinant adenoviruses were actually generated with the new technique. Furthermore, we exclusively then continued our work with the AdCD-virus. Therefore it cannot be ruled out that under other conditions the presented technique may produce other results than the conventional technique. The positive clones were processed and finally transfected into 911 cells. After harvesting recombinant adenovirus, we infected cells with AdCD and checked the expression of the Cytosin deaminase protein by Western blot. The functionality of the gene was proven by FACS analysis. We confirmed expression of the protein as well as its functionality.

## Conclusions

The presented protocol allows fast detection of recombinant clones within the AdEasy system with an easy, cost-effective technique. Therefore, this procedure is a potential alternative for screening recombinants within the AdEasy system.

## Authors' contributions

DA designed the study and was responsible for manuscript preparation, MK, IB optimised the PCR protocol, PK was responsible for manuscript preparation, MB contributed to manuscript preparation and JW was responsible for study design and manuscript preparation. All authors read and approved the final manuscript.
